# Surrogate Data Method Requires End-Matched Segmentation of Electroencephalographic Signals to Estimate Non-linearity

**DOI:** 10.3389/fphys.2018.01350

**Published:** 2018-09-27

**Authors:** Laura Päeske, Maie Bachmann, Toomas Põld, Sara Pereira Mendes de Oliveira, Jaanus Lass, Jaan Raik, Hiie Hinrikus

**Affiliations:** ^1^Centre of Biomedical Engineering, Department of Health Technologies, Tallinn University of Technology, Tallinn, Estonia; ^2^Qvalitas Medical Centre, Tallinn, Estonia; ^3^Department of Electrical and Computer Engineering, Faculty of Engineering, University of Porto, Porto, Portugal; ^4^Department of Computer Systems, Tallinn University of Technology, Tallinn, Estonia

**Keywords:** EEG, dominant frequency, alpha frequency, surrogate data, Fourier transform, segment length

## Abstract

The aim of the study is to clarify the impact of the strong cyclic signal component on the results of surrogate data method in the case of resting electroencephalographic (EEG) signals. In addition, the impact of segment length is analyzed. Different non-linear measures (fractality, complexity, etc.) of neural signals have been demonstrated to be useful to infer the non-linearity of brain functioning from EEG. The surrogate data method is often applied to test whether or not the non-linear structure can be captured from the data. In addition, a growing number of studies are using surrogate data method to determine the statistical threshold of connectivity values in network analysis. Current study focuses on the conventional segmentation of EEG signals, which could lead to false results of surrogate data method. More specifically, the necessity to use end-matched segments that contain an integer number of dominant frequency periods is studied. EEG recordings from 80 healthy volunteers during eyes-closed resting state were analyzed using multivariate surrogate data method. The artificial surrogate data were generated by shuffling the phase spectra of original signals. The null hypothesis that time series were generated by a linear process was rejected by statistically comparing the non-linear statistics calculated for original and surrogate data sets. Five discriminating statistics were used as non-linear estimators: Higuchi fractal dimension (HFD), Katz fractal dimension (KFD), Lempel-Ziv complexity (LZC), sample entropy (SampEn) and synchronization likelihood (SL). The results indicate that the number of segments evaluated as non-linear differs in the case of various non-linear measures and changes with the segment length. The main conclusion is that the dependence on the deviation of the segment length from full periods of dominant EEG frequency has non-monotonic character and causes misleading results in the evaluation of non-linearity. Therefore, in the case of the signals with non-monotonic spectrum and strong dominant frequency, the correct use of surrogate data method requires the signal length comprising of full periods of the spectrum dominant frequency. The study is important to understand the influence of incorrect selection of EEG signal segment length for surrogate data method to estimate non-linearity.

## Introduction

Non-linear dynamics is the most appropriate way to describe complex physiological systems and is therefore widely used in biomedical applications. During last decades, the interest in the theory of non-linear dynamics has increased due to raising interest in brain functioning and the necessity to understand complex dynamics of the underlying processes (Hornero et al., 2009; Rodríguez-Bermudez and Garcíıa Laencin, 2015).

The brain is assumed to function as a self-organizing complex network of interacting dynamical non-linear subsystems. Despite some cellular processes may be random and characterized by probability functions, the neural systems may exhibit rather chaotic non-linear nature. Large networks of interconnected neurons behave as self-organized large systems with local non-linear interactions (Hornero et al., 2009). The question, whether EEG signals should be looked at as a non-linear deterministic process or a linear stochastic one, is still open. Therefore, before analyzing EEG signals by non-linear methods, it is required to assess whether the non-linearity exists in the data. In case non-linearity is present, the non-linear dynamics theory could also characterize the intrinsic nature of EEG, helping to understand its dynamics, underlying brain processes and search for its physiological significance, without losing or ignoring important information (Natarajan et al., 2004). The presence of non-linearity can be confirmed by hypothesis testing.

Theiler et al. (1992) described a statistical approach for identifying non-linearity in a time series, through the surrogate data method. A surrogate data is generated from the original data by shuffling the phase spectra. Null hypothesis that data were generated by a linear process is tested by comparing non-linear statistic calculated for original and surrogate data. If the value for original data is significantly different, the null hypothesis can be rejected and non-linearity concluded. The probability that the surrogate data test will reject null hypothesis depends on the non-linear statistic used (Spasic, 2010).

Surrogate data method is widely used on EEG signals for testing the null hypothesis of linearity. There are two main purposes for surrogate data testing. The first purpose is to test whether the chosen non-linear measure captures non-linear structure in the data, which cannot be detected with spectral density function (Breakspear and Terry, 2002; Natarajan et al., 2004; Spasic, 2010; Bae et al., 2017; Orgo et al., 2017). If the data does not have any non-linear structure, a linear method could be used instead. The second purpose is to determine the statistical threshold of connectivity values in network analysis (Dimitriadis et al., 2015, 2017; Olejarczyk et al., 2017), which is being used by a growing number of studies with the method of surrogate data. However, some factors can cause misleading results for EEG signal linearity estimation. Surrogate data testing for a linear stochastic system can indicate false non-linearity in case the process is non-stationary (Timmer, 1998). A specific problem has been identified that false detection of non-linearity may occur in case the data are strongly cyclic (Stam et al., 1998; Small and Tse, 2002). The problem arises when the length of the analyzed signal segment deviates from the multiple full periods of the cyclic component in the signal.

Electroencephalographic (EEG) signal has a strong alpha frequency component in the frequency range between 9 and 11 Hz. This rhythm is most pronounced in occipital region, but is also present in central, temporal or even frontal regions. Alpha rhythm is best revealed during eyes-closed resting state. Therefore, it might be expected that due to the strong cyclic alpha component of the resting eyes-closed signal, the surrogate data method may give false results.

The aim of the study is to clarify the impact of the strong cyclic signal component on the results of surrogate data method in the case of EEG signals. In addition, the impact of segment length is analyzed. For this reason, the degree of non-linearity was found in eyes-closed resting EEG signal depending on the analyzed segment length and deviation from full period of the dominant cyclic component. Five discriminating statistics were used as non-linear estimators: Higuchi fractal dimension (HFD), Katz fractal dimension (KFD), Lempel-Ziv complexity (LZC), sample entropy (SampEn), and synchronization likelihood (SL).

## Materials and Methods

### Subjects

Eighty healthy volunteers (38 female and 42 male) aged 37.0 ± 14.5 years participated in the study. The experiments were approved by the Tallinn Medical Research Ethics Committee and were conducted in accordance with the Declaration of Helsinki. All subjects signed an informed consent.

### EEG Recordings

The EEG was recorded using Neuroscan Synamps2 acquisition system (Compumedics, Charlotte, NC, United States) from 30 electrodes, positioned according to the extended international 10–20 system. The sampling frequency was 1,000 Hz. Linked mastoids were used as a reference and electrode impedances were kept below 10 kΩ. EEG was recorded for 6 min, during which subjects were lying in a relaxed position with their eyes closed.

### Surrogate Data

Multivariate surrogate data method is used to test whether data were generated by a non-linear process (Theiler et al., 1992; Prichard and Theiler, 1994). The null-hypotheses that data were generated by a linear process and therefore data can be fully explained by a linear model, is set. Surrogate data is generated from original data. If the non-linear statistic calculated for original data significantly differs from the non-linear statistic calculated for surrogate data, null-hypothesis is rejected and non-linearity is detected.

Surrogate data is calculated from time series according to the algorithm by Prichard and Theiler (1994). Fourier transform is applied and the phase of each frequency component is independently rotated by a random degree between (0, 2π). After that, inverse Fourier transform is performed. As a result, the power spectrum and the autocorrelation function of the time series is preserved. For multivariate time series, a fixed random sequence is used to alter the phase of each frequency, ensuring linear correlations between simultaneously recorded time series.

To determine whether the value of the non-linear statistic for the original data set significantly differs from the non-linear statistics for the surrogate data, z-test is used (Breakspear and Terry, 2002):

(1) $$Z=\frac{Q_{data}-mean\;(Q_{surrogate})}{std\;(Q_{surrogate})}$$

where *Q_data_* is the non-linear statistic calculated for the original data set, *mean* (*Q_surrogate_*) is the mean and *std* (*Q_surrogate_*) is the standard deviation of linear statistics calculated for the surrogate data. In the current study, surrogate data was calculated 20 times for each data segment and the significance level of *p* < 0.05 was used. Under the null hypothesis, z-statistic is normally distributed and when | Z| >1.96 for a two-tailed test, the null hypothesis can be rejected. For data analysis, we calculated the degree of non-linearity (DEG), which we define as the percentage of segments where the null hypothesis was rejected and non-linearity was detected:

(2) $$DEG=\frac{n_{sign}}{n}\cdot 100\%$$

where *n* is the number of segments and *n_sign_* is the number of segments, where | Z| >1.96.

### Non-linear Statistics

The measures for estimation of non-linearity were selected based on two main criteria. Firstly, whereas different estimators detect various aspects of non-linearity, the applied measures should describe one of the specific features of the signals: self-similarity, dimension-based morphology, complexity, irregularity or functional connectivity. Secondly, less time-consuming methods currently widely used in EEG analysis should be represented. As a result, five non-linear methods were selected: HFD, KFD, LZC, SampEn, and SL. HFD and KFD are fractal dimension methods, LZC is a measure of complexity and SampEn is a measure of irregularity. As connectivity between neurons and synchronization of their spiking play crucial role in the brain functioning, functional connectivity measure SL, although computationally time consuming, was also selected.

The HFD evaluates the complexity and self-similarity of time series (Higuchi, 1988). It is calculated directly in the time domain, making it a simple and fast method. The HFD with a parameter *k_max_* = 8 was calculated according to the algorithm presented by Higuchi (1988).

The KFD obtains fractal dimension based on morphology, measuring the roughness of the time series (Katz, 1988). The KFD is the ratio of the length of the curve (sum of distances between two successive points), divided by the maximum distance of any point under consideration from the first point. In other words, the ratio of the total length to the straight line corresponding to the maximum distance from the first point. In addition, a scaling factor, an average of the distances between two successive points is used.

Higuchi’s and Katz fractal dimensions are the most common methods of estimating the fractal dimension of EEG signals directly in the time domain. Despite both, HFD and KFD describe the fractal dimension of EEG waveform, the behavior of the measures is different. HFD has been suggested being the most accurate, whereas KFD yields the most consistent results regarding discrimination between brain functional states (Esteller et al., 2001). Therefore, both are applied in this study.

The LZC evaluates the randomness of finite sequences (Lempel and Ziv, 1976). First, the EEG signal is transformed into a finite symbol sequence, according to a chosen threshold. Next, the sequence of symbols is analyzed from left to right. The LZC counts the number of times a new pattern is encountered and its recurrence rate for the given sequence. LZC is simple to calculate and does not need long data segments. Larger LZC values correspond to signals that are more complex. Still, the LZC strongly depends on the signal bandwidth (Kalev et al., 2015). In the current study, median value of the sequence was selected as threshold, as it is capable of coping with outliers. Next, the data was binarized (two symbols) according to the threshold. Due to artifact free sequences, selecting between median or mean is not expected to change the outcome considerably.

The SampEn measures the signal irregularity (Richman and Moorman, 2000). Signals that are more irregular give larger SampEn values. The method is quite independent of the signal length. It is suitable for analyzing short and noisy time series. The SampEn is the negative natural logarithm of the conditional probability that two sequences similar for *m* = 2 points remain similar at the next point. Parameters for the SampEn were chosen according to recommendations from previous studies (Richman and Moorman, 2000; Lake and Moorman, 2010): the embedding dimension *m* = 2 and the tolerance *r* = 0.2 *SD*, where *SD* is the standard deviation of the sample.

The SL is a non-linear measure of functional connectivity (Stam and Van Dijk, 2002). The SL estimates dynamical interdependencies between simultaneously recorded time series using Takens’ theorem (Takens, 1981) of reconstructing EEG signals into state space. The calculation of the SL is more thoroughly explained in the article by Stam and Van Dijk (2002). The SL parameters were calculated according to the formulas presented in the paper by Montez et al. (2006) with respect to the time-frequency content of the signal. Therefore, the following parameters were used: the embedding lag *L* = 7, the embedding dimension *m* = 136, the number of recurrences *n_rec_* = 10, the fraction of recurrences *p_ref_* = 0.01, window *W*_1_ = 2000 and window *W*_2_ = 2999. Such selection of the parameters ensures that the time-frequency characteristics of the signals are fully taken into account. Therefore, small alterations in these parameters are not expected to change the results of surrogate data method significantly.

### Data Processing

Data processing was done in MATLAB (The Math-works, Inc.) using signal processing toolbox. Signals were digitally filtered (1–45 Hz) using zero-phase Butterworth filter and re-referenced according to the reference electrode standardization technique (REST) (Yao, 2001). Signals were divided into 5.3-s segments. Data were visually inspected and segments with artifacts were not analyzed.

Surrogate data method makes an assumption of stationarity. We conducted two stationarity tests: the Kwiatkowski–Phillips–Schmidt–Shin (KPSS) and the Phillips–Perron (PP) test and no non-stationarity was detected.

#### Dependence on the Segment Length Increment for Alpha Component

The aim of the current section was to determine how DEG depends on the segment length increment. For that purpose, the length of the segment was gradually incremented from an integer number of alpha periods by 2 ms. Therefore, the length of each segment was determined as:

(3) l=kT+Δt, Δt=0,  2,  4,…, 108ms,

where *k* is an integer, *T* is the period of alpha frequency component and Δ*t* is the segment length increment. The first segment was approximately 5 s, starting and ending at the alpha peak amplitude (Δ*t* = 0) – consisting of an integer number of alpha periods. Therefore, the exact length of the first segment depended on the alpha period. The second segment started at the same position as the first one, but ended 2 ms later (Δ*t* = 2). Finally, the length of the last segment (Δ*t* = 108) was approximately 5.1 s. For most subjects, the length of the last segment corresponds to *l* = (*k +* 1)*T* – again an integer number of alpha periods. As there were 62 data segments for a subject, we repeated the incrementation procedure for each of the 62 data segments and DEG was calculated according to formula (2) for each Δ*t* = 0, 2, 4, …, 108 ms, where *n* = 62.

Alpha peaks were found by zero-phase filtering signals into alpha frequency band (7.5–13 Hz) using Butterworth filter and peaks were indicated by local maxima. The channel O1 was chosen for processing, because of the highest average alpha power. After finding positions of alpha peaks in channel O1, whole frequency band (1–45 Hz) was used for calculating DEG. The dependence on Δ*t* was found for five different non-linear parameters: HFD, KFD, LZC, SampEn and SL. As SL is calculated between two channels, O1 and O2 were used.

#### Dependence on Channel

In different channels, the amount of alpha power, the strong cyclic component, differs. This component is most pronounced in occipital region, but is also present in other regions. To analyze the dependence on the EEG channel, three channels were chosen according to average mean alpha power: O1 with the highest alpha power, C3 with average alpha power and T7 with the lowest alpha power. In addition to O1, analysis for C3 and T7 were conducted in accordance to 2.5.1, whereas HFD was used as a non-linear measure.

#### Dependence on the Segment Length Increment for Different Frequency Components

It is well known that alpha is the dominant frequency during eyes-closed resting state EEG recordings, especially in posterior areas. However, it is important to clarify, whether the surrogate data method is also affected by the cyclic component of other EEG frequency bands. For that purpose, the analysis in 2.5.1 was repeated using HFD, but the segments beginning and the segment length increment have been matched to the following frequencies: delta (1–1.5 Hz; Δ*t* = 0, 20, …, 1000), theta (4–8 Hz; Δ*t* = 0, 3, …, 126) and beta (13–30 Hz; Δ*t* = 0, 1, …, 46). For better comparison, the results for alpha component (7.5–13 Hz; Δ*t* = 0, 2, …, 108) are also presented.

#### Dependence on Segment Length

While incrementing the segment by Δ*t*, the overall segment length was almost the same, between 5 and 5.1 s. To analyze the dependence on the segment length, the data were divided into substantially different segment lengths: around 5, 10, 15, and 20 s. Each segment started from alpha peak and ended with alpha peak, consisting of an integer number of alpha periods. Each subject had 10 segments of each segment length, whereas *n* = 10 in formula (2). DEG was calculated for each subject and segment.

### Data Processing

The observations of DEG were obtained for each subject. The dependence on the Δ*t* and the segment length were statistically evaluated using one-way analysis of variance (ANOVA) with the significance level of *p* < 0.05. To correct for the problem of multiple comparisons, Bonferroni correction was used by adjusting the *p*-value *p* = *p*/*m*, where *m* is the number of comparisons.

## Results

Average DEG values for end-matched segments according to alpha frequency (Δ*t* = 0) are presented in **Table 1**. The percentage of segments where non-linearity was detected varies significantly depending on the non-linear measure. KFD indicated the highest degree of non-linearity: the KFD value was significantly changed in 99% of segments, while LZC revealed non-linearity only in 0.4% of the segments.

**Table 1:** The degree of non-linearity at alpha peak.

	DEG, %
HFD	46.1
KFD	99.1
LZC	0.4
SampEn	81.5
SL	3.9

### Dependence on the Segment Length Increment for Alpha Component

**FIGURE 1:**
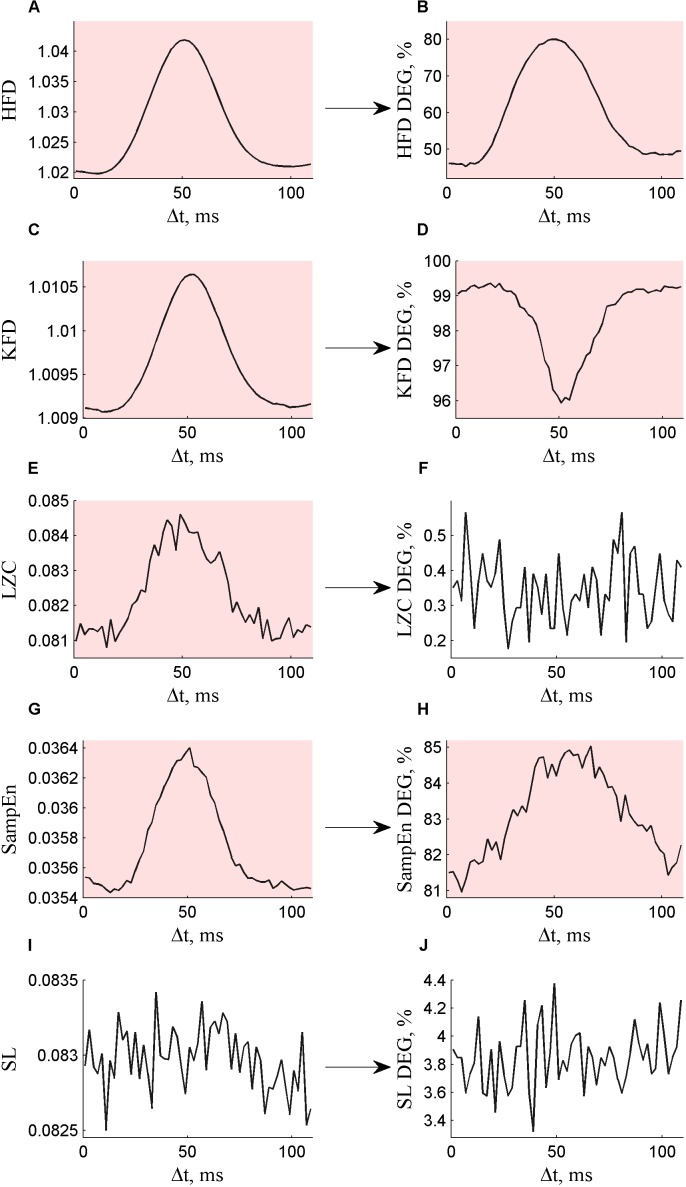
Non-linear measures **(A)** HFD, **(C)** KFD, **(E)** LZC, **(G)** SampEn, and **(I)** SL calculated for surrogate data depending on the segment length increment Δ*t* are presented on the left. The degree of non-linearity DEG depending on the segment length increment Δ*t* for **(B)** HFD DEG, **(D)** KFD DEG, **(F)** LZC DEG, **(H)** SampEn DEG, and **(J)** SL DEG are presented on the right. Statistically significant results are indicated with a pink background.

The calculated DEG values for HFD, KFD, LZC, SampEn and SL in alpha frequency band are presented in **Supplementary Datasets 1–5**. We conducted ANOVA to analyze whether the segment length increment Δ*t* influences the results of surrogate data method. ANOVA (*p* < 0.05/5) yielded statistically significant results for every non-linear statistic that indicated non-linearity (*DEG* > 5%): HFD DEG (**Figure 1B**), KFD DEG (**Figure 1D**) and SampEn DEG (**Figure 1H**). For example, when Δ*t* = 0, then HFD DEG was 46.1%, but Δ*t* = 50 (corresponding to half alpha period) resulted in HFD DEG 80.0%. LZC DEG (**Figure 1F**) and SL DEG (**Figure 1J**) did not depend on the Δ*t*.

In order to understand the DEG results presented in **Figure 1**, we can consider the values of non-linear measures calculated for original and surrogate data, according to which DEG was calculated. Incrementing the segment length to Δ*t* = 50 increased the values calculated for surrogate data for all five non-linear measures, but the increase was statistically significant only for HFD (**Figure 1A**), KFD (**Figure 1C**), LZC (**Figure 1E**), and SampEn (**Figure 1G**). Since HFD and SampEn calculated for surrogate data were significantly increased compared to the values calculated for original data, this resulted in an increase also in DEG (**Figures 1B,H**). However, KDF for surrogate data was significantly decreased compared to KFD for original data, resulting in a decrease in DEG (**Figure 1D**). Although LZC calculated for surrogate data was also influenced by segment length increment (**Figure 1E**), LZC was similar for original and surrogate data, yielding low DEG values, resilient to segment length increment (**Figure 1F**).

### Dependence on Channel

**FIGURE 2:**
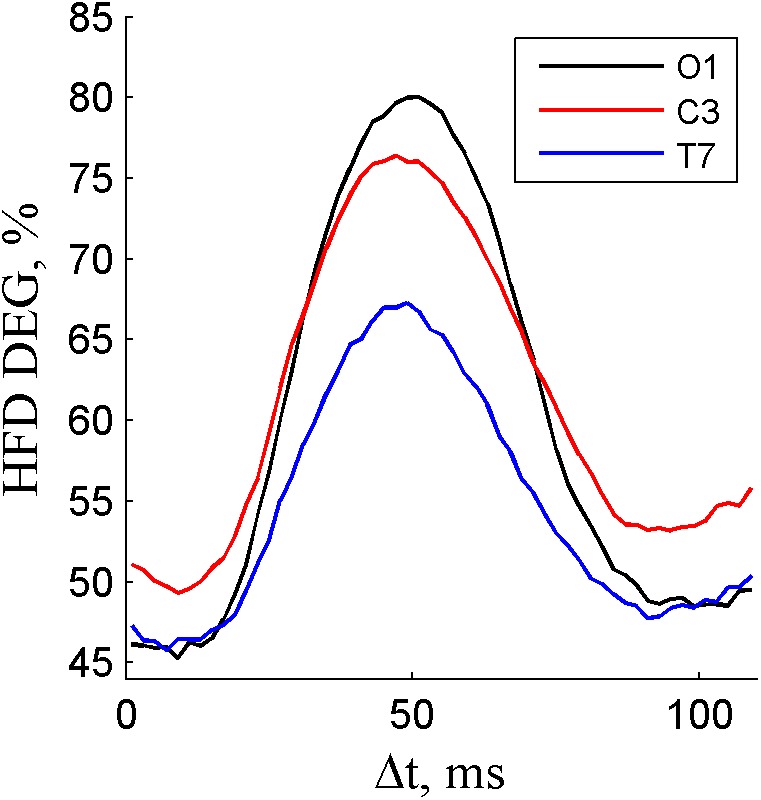
The degree of non-linearity DEG depending on the segment length increment Δ*t* for alpha component in channels O1, C3, and T7.

The calculated HFD DEG values for channels O1, C3 and T7 are presented in **Supplementary Datasets 1**, **6, 7**. According to ANOVA (*p* < 0.05/3), HFD depended on the Δ*t* for all studied channels. The deflection in DEG was the largest in channel O1, followed by C3 and T7 (**Figure 2**). These results are in accordance with the amount of spectral alpha power in those channels.

### Dependence on the Segment Length Increment for Different Frequency Components

**FIGURE 3:**
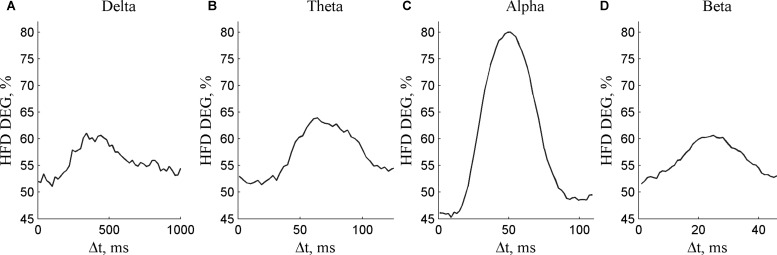
The degree of non-linearity DEG depending on the segment length increment Δ*t* for **(A)** delta, **(B)** theta, **(C)** alpha, and **(D)** beta frequency components.

The calculated HFD DEG values for delta, theta, alpha and beta frequency components are presented in **Supplementary Datasets 1**, **8–10**. According to ANOVA (*p* < 0.05/4), HFD depended on every calculated cyclic component (**Figure 3**). The difference between maximum and minimum DEG for different Δ*t* was the largest for alpha component (80.0% – 45.3% = 34.7%), followed by theta (63.9% - 51.4% = 12.5%), delta (61.1%-51.1% = 10.0%), and beta component (60.6% - 51.6% = 9.1%).

### Dependence on Segment Length

The influence of segment length (Δ*t* = 0) on DEG was investigated for five non-linear measures: HFD, KFD, LZC, SampEn, and SL (**Supplementary Dataset 11**). The results are presented in **Table 2**. According to ANOVA (*p* < 0.05/20), DEG depended on the segment length for HFD, SampEn, and SL (marked with ^∗^ in **Table 2**). The results for 5-s segments are slightly different from the results in **Table 1**, because smaller number of segments were used.

**Table 2:** The degree of non-linearity at different segment lengths (^∗^*p* < 0.05).

	DEG, %			
**Segment length**	**5 s**	**10 s**	**15 s**	**20 s**
HFD	45.7^∗^	41.0^∗^	36.4^∗^	34.0^∗^
KFD	99.9	100	100	100
LZC	0.4	0.6	0.2	0.4
SampEn	82.0^∗^	95.4^∗^	98.5^∗^	99.4^∗^
SL	4.4^∗^	6.5^∗^	7.0^∗^	9.1^∗^

## Discussion

The aim of the study was to clarify the impact of the strong cyclic EEG signal component on the results of surrogate data method by Theiler et al. (1992). In addition, the impact of segment length was analyzed. The major finding of the study was that if the EEG segment does not contain an integer number of full alpha periods, the values calculated for surrogate data may be significantly altered, resulting in a false rejection of linearity. To the best of our knowledge, similar results have not been reported earlier.

Previous studies have shown that false detection of non-linearity may occur when the data are strongly cyclic (Stam et al., 1998; Small and Tse, 2002). However, the influence of this problem on EEG signals was not previously known. Although surrogate data method is widely used for EEG analysis (Breakspear and Terry, 2002; Natarajan et al., 2004; Spasic, 2010; Dimitriadis et al., 2015, 2017; Bae et al., 2017; Olejarczyk et al., 2017), the cyclic behavior of dominant frequency component is not considered in segmentation. The current study shows the importance of segmenting data according to the alpha component for eyes-closed resting state EEG.

Our results demonstrate remarkable non-monotonic changes in the degree of non-linearity of EEG signals with the fine tuning of the segment length within a period of dominant EEG signal frequency for every non-linear statistic that indicated non-linearity (DEG > 5%): HFD (**Figure 1B**), KFD (**Figure 1D**), and SampEn (**Figure 1H**). The changes in the degree of non-linearity are caused by the changes in the non-linear measures calculated for surrogate data (**Figures 1A,C,G**), whereas the measures calculated for original data have no remarkable dependence on so small alteration of segment length. The impact of segment length tuning on the results of surrogate data method is maximal when the segment length contains an odd number of half-periods of the dominant frequency (**Figure 1**). The phenomenon can be explained by spectral leakage in the discrete Fourier transform while deriving the surrogates, as discrete Fourier transform assumes periodic signals. Thornhill (2005) showed that even a small spectral component other than that at the dominant frequency could be interpreted as non-linearity and causes false detection of non-linearity for sine waves. However, they showed that pseudoperiodic data with weaker cyclic behavior were more robust to small end-mismatches. These results are in accordance with the results in the current study. Moreover, the current study proves that the cyclic behavior of EEG has a strong influence on non-linear measures calculated for surrogate data for large end-mismatch.

Two measures, LZC (**Figure 1F**) and SL (**Figure 1J**), did not detect significant non-linearity (DEG < 5%). In the case of LZC, the possible reason is that the measure is highly sensitive to low frequency EEG component in binarization due to its high amplitude values. The non-linearity, if contained in the low amplitude high frequency activity, gets overlooked in the process of binarization and is not detected by the measure. SL did not detect non-linear coupling, indicating that SL does not necessarily give significantly more information compared to similar linear functional connectivity measures.

The level of alterations caused by fine tuning within a period of dominant frequency differs at different non-linear discrimination measures. The degree of linearity changes about two-fold with HFD, is much lower with KDF and SampEn and becomes insignificant with LZC and SL. The different impact of fine tuning of segment length within a period of dominant frequency can be explained by different sensitivity of various non-linear measures to a small additional spectral component introduced by the deviation of the segment length from a full period. The problem can be solved by selecting the start and end of the segment by matching the period of the strong cyclic component. A segment end-matching can be performed by selecting a segment length equal to integer number of full periods of the dominant frequency (Stam et al., 1998). In addition, Small et al. (2001) suggested an alternative surrogate data method: pseudo-periodic surrogate (PPS) algorithm. However, PPS is not applicable to data where the non-linearity of interest is distortion of the periodic waveform (Thornhill, 2005).

The dependence of the degree of non-linearity on the segment length increment from full alpha periods has the maximal value for alpha frequency component (**Figure 3**). The alteration of the degree of non-linearity with the dominant frequencies in delta, theta or beta bands are less critical. The possible reason is the structure of EEG signal with a dominant alpha frequency. The minimum DEG value in **Figure 3** is the smallest for alpha frequency component. These results show that the synchronization of the fine tuning of the segment length should be performed with the dominant frequency component to decrease the amount of false positive surrogate data results.

The dependence of the degree of non-linearity on the segment length increment from full period of dominant EEG frequency is evident in various EEG channels (**Figure 2**). As expected, the impact is stronger in the EEG channels with higher alpha content (O) and weaker in channels with lower alpha content (T). The influence of segment end-mismatch on other channels also mostly depends on the spectral alpha power and lies between the obtained results of O1 and T7 (**Figure 2**). The results may also be influenced by an additional strong frequency component (channel C3 in **Figure 2**), but the dominant frequency component should be taken into account in segment end-matching.

The degree of linearity estimated at an integer number of alpha periods (**Tables 1**, **2**) shows that the degree of non-linearity varies for different non-linear measures. Different sensitivity to surrogate data method has also been reported by other author (Spasic, 2010) when comparing HFD and third order correlation. Our results suggest that HFD, KFD, and SampEn were more sensitive to non-linearity, while SL and LZC values changed significantly in less than 5% of segments for 5-s segments. In this case, SL has been calculated between O1 and O2 channels. The results can vary for different channel pairs, but Orgo et al. (2017) found that for SL 5-s segments, the average degree of non-linearity over all channel pairs was similar to that in our current study (6.1% compared to our 4.4%). In addition, the degree of non-linearity estimated in the current study is close to the results reported by Breakspear and Terry (2002), who detected statistically significant evidence of non-linear interactions in 4.8% of the 2.048-s segments of eyes-closed resting state EEG.

The findings presented in **Table 2**, indicating changed non-linearity with increased segment length, are in principle in accordance with the results reported by other research groups (Olbrich et al., 2003; Sun et al., 2012; Orgo et al., 2017). Olbrich et al. (2003) have reported the dependence of rejection of the null hypothesis between natural and surrogate data in sleep EEG on the length of the analyzed segment. They suggested that the increase of evaluated non-linearity with the segment length might occur because of the increasing non-stationarity of the longer time series. In the current study, KPSS and PP test did not reveal any non-stationarity. Sun et al. (2012) have made a conclusion that the length of signal segment for analysis of 3–16 periods is sufficient for detecting non-linearity in the case of EEG phase synchronization. However, in the current study we showed that the results of evaluation of non-linearity vary even with the segment lengths of more than 100 periods. Orgo et al. (2017) were the first to compare the degree of EEG non-linear coupling in different frequency bands and segment lengths, during eyes-closed resting state. Their results showed that the degree of non-linear coupling increased with the length of the segment, and it was most dominant in total, alpha, beta and theta frequency bands.

## Conclusion

The results of the performed study show that the selection of a proper segment length in evaluating non-linearity of EEG signals with surrogate data method is critical to assure the reliability of evaluation. The results of performed calculations demonstrate that false rejection of linearity occurred with surrogate data method when an EEG segment did not contain an integer number of full alpha periods using HFD, KFD, or sample entropy. LZC and SL did not detect significant non-linearity and were therefore not influenced by segment end-mismatch. The major novel finding is that the correct estimation of non-linearity with surrogate data method requires a segment length comprising of full periods of the spectrum’s dominant frequency component. In addition, the degree of non-linearity estimated with HFD, sample entropy and synchronization likelihood significantly changed with the segment length.

## Data Availability Statement

The datasets analyzed in this study can be found as a xlsx file in the supplement.

## Author Contributions

LP and MB designed the study and conducted EEG recordings. LP processed the data. LP, MB, and HH analyzed and interpreted the results and wrote the manuscript. TP, SdO, JL, and JR contributed to discussion of results and writing the manuscript. All authors revised and approved the final manuscript.

## Conflict of Interest Statement

The authors declare that the research was conducted in the absence of any commercial or financial relationships that could be construed as a potential conflict of interest.
